# Stem Cell-Derived Extracellular Vesicles and Immune-Modulation

**DOI:** 10.3389/fcell.2016.00083

**Published:** 2016-08-22

**Authors:** Jacopo Burrello, Silvia Monticone, Chiara Gai, Yonathan Gomez, Sharad Kholia, Giovanni Camussi

**Affiliations:** Stem Cell Laboratory, Department of Medical Sciences, University of TorinoTorino, Italy

**Keywords:** extracellular vesicles, exosomes, stem cells, immune system, immuno-modulation

## Abstract

Extra-cellular vesicles (EVs) are bilayer membrane structures enriched with proteins, nucleic acids, and other active molecules and have been implicated in many physiological and pathological processes over the past decade. Recently, evidence suggests EVs to play a more dichotomic role in the regulation of the immune system, whereby an immune response may be enhanced or supressed by EVs depending on their cell of origin and its functional state. EVs derived from antigen (Ag)-presenting cells for instance, have been involved in both innate and acquired (or adaptive) immune responses, as Ag carriers or presenters, or as vehicles for delivering active signaling molecules. On the other hand, tumor and stem cell derived EVs have been identified to exert an inhibitory effect on immune responses by carrying immuno-modulatory effectors, such as transcriptional factors, non-coding RNA (Species), and cytokines. In addition, stem cell-derived EVs have also been reported to impair dendritic cell maturation and to regulate the activation, differentiation, and proliferation of B cells. They have been shown to control natural killer cell activity and to suppress the innate immune response (IIR). Studies reporting the role of EVs on T lymphocyte modulation are controversial. Discrepancy in literature may be due to stem cell culture conditions, methods of EV purification, EV molecular content, and functional state of both parental and target cells. However, mesenchymal stem cell-derived EVs were shown to play a more suppressive role by shifting T cells from an activated to a T regulatory phenotype. In this review, we will discuss how stem cell-derived EVs may contribute toward the modulation of the immune response. Collectively, stem cell-derived EVs mainly exhibit an inhibitory effect on the immune system.

## Introduction

**Figure 1:**
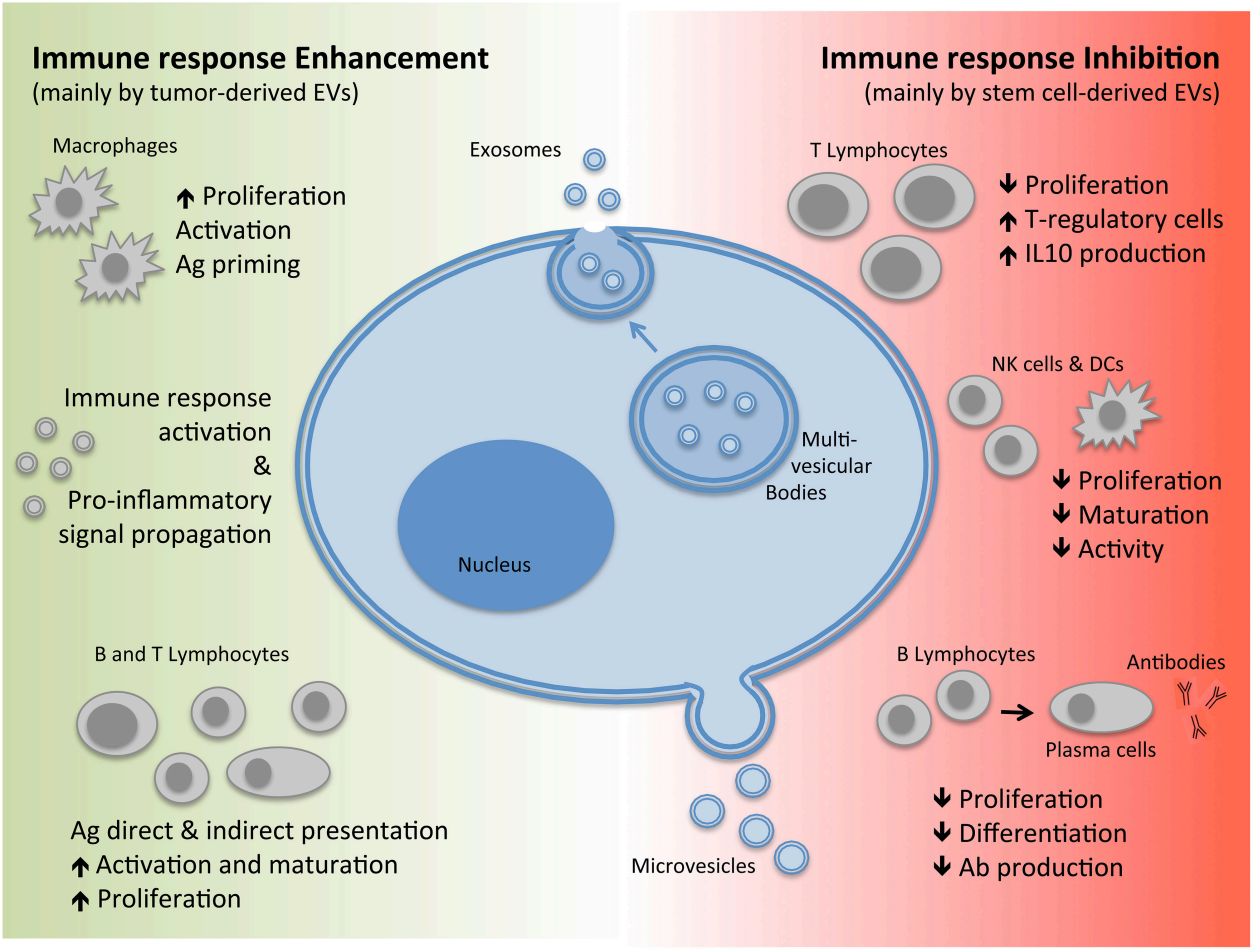
**Biogenesis of EVs and their immuno-modulatory effects**. EVs, Extracellular Vesicles; Ag, Antigen; IL, Interleukin; Ab, Antibodies.

Extra-cellular vesicles (EVs) are bilayer membranal structures released by cells as a means of transferring their content to and from other cells, now acknowledged as a novel mechanism of intercellular communication. These vesicles comprise of both membrane and cytoplasmic components including: proteins, nucleic acids, and other active molecules and can be sub classified according to size, biogenesis, and composition into exosomes or microvesicles (also defined as shedding vesicles; Théry et al., 2009; EL Andaloussi et al., 2013; Yáñez-Mó et al., 2015; see Figure 1).

The term “microvesicle” generally refers to both vesicles released by healthy cells as well as pre-apoptotic vesicles. They are commonly heterogeneous in size ranging from 50 to 1000 nm in diameter depending on the state of the cell during release by either direct shedding or budding from the plasma membrane (Figure 1). Exosomes, on the other hand, are generated through an invagination process of the endosomal membrane of multi-vesicular bodies (MVBs) found within the cells forming vesicles. This is followed by fusion of the MVBs with the plasma membrane leading to exocytosis and release of exosomes which are homogenous in size with a diameter ranging from 40 to 200 nm (Figure 1; Yáñez-Mó et al., 2015). Since exosomes and microvesicles share several molecular and functional characteristics and are released concomitantly by the same cell types, the term EVs will be used to collectively indicate the two subtypes throughout this review.

EVs carry a plethora of molecules that influence their mode of action. These include a variety of receptors, adhesion molecules, proteins involved in cell trafficking, and/or intra-cellular signal transduction, cytoskeletal proteins, cytoplasmic enzymes, cytokines, chemokines, as well as cell-specific antigens (Ags). Moreover, they are also enriched with a range of nucleic acids, including mRNA, long non-coding RNAs, microRNAs (miRNA), and even extra-chromosomal DNA (Robbins and Morelli, 2014; Yáñez-Mó et al., 2015). The content carried however may vary depending on the type of cell and the state of activation. Once released, these EVs may interact with neighboring cells or diffuse through and circulate in the bloodstream or other organic fluids such as: breast milk, semen, saliva, urine, and sputum. Their state of ubiquity makes them important effectors of cell-to-cell communication, which may occur in a paracrine, autocrine, exocrine, and or endocrine manner (Yáñez-Mó et al., 2015).

Virtually all cell types have the ability to release EVs including cells of the immune system such as: macrophages, antigen presenting cells (APCs), dendritic cells (DCs), B and T cells (both CD4^+^ T helper and CD8^+^ cytotoxic T lymphocytes), natural killer cells (NKs), as well as cells complementing the immune system such as platelets, mast cells, fibroblasts, epithelial cells, and stem cells. Recently, there has been enough growing evidence to suggest that EVs play an essential role in immuno-modulation (Robbins and Morelli, 2014), influencing both the activation and suppression of the immune response. Furthermore, a role of EVs has also been implicated in inflammation, autoimmune, infectious, and cancer diseases (Yáñez-Mó et al., 2015). This review will however focus on the interactions of stem-cell derived EVs with the immune system.

## Regulation of immune response by EVs

The immune system can be divided into two branches: the innate immune response (IIR)—an evolutionary conserved system common to all multicellular organisms, and the acquired or adaptive immune response (AIR)—an exclusive feature developed in vertebrates (Sirisinha, 2014).

Within the IIR system, EVs act as paracrine messengers, allowing the propagation of pro-inflammatory signals (Mastronardi et al., 2011; Wang et al., 2011; Prakash et al., 2012; Cloutier et al., 2013). However, they have also been reported to contribute as negative regulators of the inflammatory response primarily by carrying molecules such as Transforming Growth factor-β (TGF-β), and other immuno-suppressive mediators (Gasser and Schifferli, 2004). The role of EVs in the regulation of IIR is complex and has not yet been fully elucidated. Many studies so far have investigated the composition and function of EVs from innate immune cells cultured *in-vitro*. For instance, macrophages treated *in vitro* with EVs isolated from cells infected with *Mycobacterium tuberculosis* released cytokines and chemokines that contributed toward the activation of the immune response (Walters et al., 2013). On the other hand, macrophages infected with the Leishmania parasite secreted EVs enriched with the Leishmania surface protein gp63, which down-regulated the inflammatory response, favoring parasite invasion (Hassani and Olivier, 2013).

Whereas, IIR is a non-specific first line of defense against microbial pathogens and other tissue injuries, AIR is a specific response induced after Ag recognition by adaptive immune cells followed by activation and clonal expansion of immune cells carrying the recognized Ag-specific receptors (Schenten and Medzhitov, 2011; Zhang et al., 2014). In this setting, EVs may act not only as Ag carriers (since they may transfer bacterial, viral, and tumoral components to APCs; O'Neill and Quah, 2008; Walker et al., 2009; Testa et al., 2010), but also as modulators of direct and indirect Ag presentation. Furthermore, this property of EVs to carry Ags from parental cells can allow them to act as reporters of foreign agents in the organism both for the host immune system as well as from a diagnostic point of view (Yáñez-Mó et al., 2015). For example, tumor-derived EVs carry tumor-Ags, which can be taken up and processed by DCs and then cross-presented to tumor-specific cytotoxic T-lymphocytes (CTLs; Wolfers et al., 2001; Andre et al., 2002). This has been demonstrated for EVs isolated from ascites of tumoral patients as well as other tumoral cell lines (Wolfers et al., 2001; Andre et al., 2002; Morelli et al., 2004). This hypothesis is supported by the fact that vaccination of mice with tumor peptide-pulsed DC-derived EVs induces a potent CD8^+^ T cell-mediated anti-tumoral effect (Wolfers et al., 2001). On the basis of these findings, it can be speculated that tumor-derived EVs carry tumor-specific Ags and that they could be used to stimulate or inhibit the immune anti-tumoral surveillance (Robbins and Morelli, 2014). In this regard, ongoing studies are exploring their potential role in the field of anti-tumor vaccination, as reviewed by Kunigelis et al. (Kunigelis and Graner, 2015). Furthermore, APC-derived EVs can also act as “Ag-presenting vesicles” for *in vitro* T-cell clones (Théry et al., 2002; Muntasell et al., 2007; Nolte-'t Hoen et al., 2009), however this activity appears to be 10–20 times less efficient to that of corresponding APCs probably due to: the small size, vesicle diffusion, and limited number of MHC molecules per vesicle (Zitvogel et al., 1998; Vincent-Schneider et al., 2002; Qazi et al., 2009).

Many recent studies on EVs have focused on the dichotomic effects they have on the immune system (see Figure 1). There are studies that have reported that EVs are able to promote the immune response by carrying foreign Ags (Bhatnagar and Schorey, 2007; Robbins and Morelli, 2014) as well as inflammatory cytokines (Pizzirani, 2007; Zuccato et al., 2007) and therefore also play a role in mediating chronic inflammatory and autoimmune diseases. For instance, EVs derived from synovial fluid of patients with rheumatoid arthritis (RA) have higher levels of TNF-alpha compared to healthy controls (Zhang et al., 2006). Furthermore, these EVs are able to delay activated T-cell mediated cell death, thereby contributing to the pathogenesis of RA (Zhang et al., 2006). Similarly, EVs isolated from broncho-alveolar fluid of patients with sarcoidosis display pro-inflammatory activity (Qazi et al., 2010). On the other hand, EVs can also have the opposite effect, mediating immuno-suppression. For instance, EVs derived from stimulated T cells are found to be enriched with major histocompatibility complex (MHC) molecules, T-cell receptors (TCR), APO2 ligand, Fas ligand (FasL), and Natural-Killer Group-2 Member-D receptor (NKG2D), which have the ability to not only inhibit Natural killer cell (NK) cytotoxicity but also promote apoptosis of T-cells and down-regulation of Ag processing by APCs (Monleón et al., 2001; Busch et al., 2008; Xie et al., 2010; Hedlund et al., 2011). Although, the function of EVs to either promote or suppress the immune response depends on the type of cell they are released from, including tumors, those derived from stem cells have mainly demonstrated suppressive properties.

## Involvement of stem cell-derived EVs in immuno-modulation

Advances in stem cell (SC) technology have opened interesting perspectives in the field of translational medicine in terms of regenerative and therapeutic strategies. Classically, stem cells can be classified into two main categories: embryonic stem cells (ESCs) and adult (or tissue) SCs (Nawaz et al., 2016).

ESCs are pluripotent cells that have the ability to differentiate into cells from any of the three embryonic germ layers: mesoderm, ectoderm, and endoderm. Furthermore, they can self-renew and proliferate indefinitely. As they are derived from embryos at 5–6 days post conception; their use is fairly limited due to ethical concerns on availability as well as for the risk of forming teratomas (Alvarez et al., 2012; Nawaz et al., 2016). Another sub type of SCs is the recently described induced pluripotent stem cells (iPS) which are adult cells that are engineered by manipulating the expression of certain genes to induce re-programming of the cell back to a pluripotent state (Takahashi and Yamanaka, 2006; Alvarez et al., 2012).

Adult stem cells are cells located in fetal and adult tissues; they have limited differentiation capabilities compared to ESCs, and are involved in the maintenance of cell turnover and tissue repair. Nomenclature of adult SCs depends mainly on the source of origin for example: SCs from the bone marrow are known as hematopoietic stem cells and mesenchymal stem/stromal cells (MSCs), and SCs from the nervous system as neural stem cells etc. (Alvarez et al., 2012).

The biological effect of SCs partly depends on a paracrine mechanism whereby, secreted factors including: growth factors, miRNAs, and EVs, influence stem cell regeneration and differentiation as well as mediate crosstalk to and from local and distant tissues (Lai et al., 2010; Zanotti et al., 2013; Wang et al., 2015a). SC-secreted factors have been reported to exhibit several properties such as: anti-apoptotic and angiogenic effects, cell-mobilization, maintenance of homeostasis as well as immune-modulation amongst others (Burdon et al., 2011; Tsuji and Kitamura, 2015). Furthermore, apart from secreted factors, stem cell-derived EVs have also been reported to mimic the effect of SCs after internalization by target cells (injured cells in the context of regeneration), mainly through receptor–ligand mediated interactions and/or direct fusion, leading to horizontal transfer of proteins and nucleic acids (including mRNA and non-coding RNA; Ratajczak et al., 2006a; Valadi et al., 2007; Camussi et al., 2010).

Many authors in the recent years have focused their research in studying the role of EVs (from various cellular origins) in immuno-modulation, however, very little studies have been reported that address the role of SC-derived EVs in the immune system particularly on IRR and AIR. Table 1 shows references of studies that have reported potential effectors identified within EVs that exhibit immuno-modulatory properties. The reason for such limited studies in the literature may partly be due to the fact that the role of SC-EVs as potential immune-modulatory effect is still novel (Nawaz et al., 2016) and partly due to the reporting of controversial results. Nevertheless, in order to elucidate the novel role(s) of SC-EVs on the immune system as well as to clarify the reported controversies in the results, further studies in this subject are only warranted.

**Table 1:** **Potential EV-derived effectors reported to play a role in immuno-modulation**.

**Effector**	**Type**	**Cell source**	**Function**	**References**
Nanog	Transcriptional factor	ESCs	Tumoral cells immune-escape	Ratajczak et al., 2006b; Yáñez-Mó et al., 2015
Oct-4	Transcriptional factor	ESCs	Innate immune suppression	Ratajczak et al., 2006b; Lee et al., 2012
HoxB4	Transcriptional factor	ESCs	Through WNT signaling affects DCs maturation and T-cells proliferation, differentiation, and activation	Ratajczak et al., 2006b; Staal et al., 2008
Rex-1	Transcriptional factor	ESCs	Through PIP3 signaling stimulate innate immune response and neutrophils activity	Weiner, 2002; Ratajczak et al., 2006b
Oct4	mRNA	ESCs	Innate immune suppression	Katsman et al., 2012; Noh et al., 2012
Sox2	mRNA	ESCs	Initiate innate response against microbial infection through neutrophils activation	Katsman et al., 2012; Xia et al., 2015
CD81 CD9	Membrane protein	iPS	Immune system cell adhesion, motility, activation, and signal transduction	Levy et al., 1998; Hu et al., 2015
miR-21	miRNA	iPS	Inhibiting effect on granulopoiesis	O'Connell et al., 2010; Wang et al., 2015b
*TGF-β1*	*Cytokine*	*MSCs*	*Maintain tolerance through regulation of lymphocyte proliferation and survival*	Bruno et al., 2015
*IL-6*	*Cytokine*	*MSCs*	*Interfere effect in DCs maturation*	Djouad et al., 2007
*IL-10*	*Cytokine*	*MSCs*	*Suppress T lymphocytes proliferation*	Hwu et al., 2000
*PGE-2*	*Prostaglandin*	*MSCs*	*Inhibit NKs activity and proliferation*	Aggarwal and Pittenger, 2005; Spaggiari et al., 2006
			*Suppress T lymphocytes proliferation*	
			*Interfere effect in DCs maturation*	
*IDO*	*Enzyme*	*MSCs*	*Inhibit NKs activity and proliferation*	Hwu et al., 2000
*miR-155 miR-146*	*miRNA*	*MSCs*	*miR-155 promotes, while miR-146 reduce, inflammatory reaction in mice*	Di Trapani et al., 2016
*HGF*	*Growth factor*	*MSCs*	*Suppress T lymphocytes proliferation*	Di Nicola et al., 2002

ESCs, Embryonic Stem Cells; iPS, induced Pluripotent Stem Cells; MSCs, Mesenchymal Stromal/Stem Cells; IL, Interleukin; TGF, Transforming Growth Factor; PG, Prostaglandin; IDO, Indoleamine 2,3-dioxygenase; HGF, Hepatocyte Growth Factor. In Italic, EVs-carried effector for which it has been directly demonstrated the immuno-modulatory effect.

Ratajczak et al. first reported that EVs released by ESCs might modulate the phenotype of target cells, supporting self-renewal of hematopoietic progenitors and multi-potency by transfer of growth factors and messenger-RNA (mRNA) (Ratajczak et al., 2006b). Subsequent studies confirmed this horizontal transfer of genetic material in other contexts, such as endothelial progenitor induced angiogenesis and modulation of bone-marrow cell phenotype by EVs released by injured tissues (Deregibus et al., 2007; Aliotta et al., 2010). Furthermore, ESC-derived EVs may not only contribute to cell-fate determination but also to tissue repair and various other physio-pathological processes, but their role as immuno-modulators has not yet been fully elucidated. Nonetheless, ESC-derived EVs have been reported to carry transcriptional factors and mRNAs such as: Nanog, octamer-binding transcription factor 4 (Oct-4), HoxB4, Rex-1, Oct4, and Sox2 that have immuno-modulatory properties (see Table 1). For instance, Nanog has been described in tumor cell immuno-escape (Ratajczak et al., 2006b; Noh et al., 2012), Oct-4 in the inhibition of IIR (Ratajczak et al., 2006b; Lee et al., 2012), and HoxB4 in impairment of DC maturation, and T-cell proliferation through a WNT-mediated mechanism (Ratajczak et al., 2006b; Staal et al., 2008). Whereas, Rex-1 and Sox2 have been shown to stimulate IIR (Weiner, 2002; Ratajczak et al., 2006b; Katsman et al., 2012; Xia et al., 2015). However, a direct role of ESC-derived EVs on the immune system through the above described effectors has yet to be reported. Similar to ESCs, little is known about the functions of iPS-derived EVs on the immune system. The available studies show that membrane proteins, such as CD81 and CD9, and miR-21 are carried by iPS-derived EVs and that these effectors may affect the immune response by regulating: cell adhesion, motility, activation and signal transduction (see Table 1; Levy et al., 1998; O'Connell et al., 2010; Hu et al., 2015; Wang et al., 2015b).

Amongst adult SCs, MSCs are certainly the most studied type (Pittenger et al., 1999; Bruno et al., 2015). In addition to regenerative effects in animal models (Syed and Evans, 2013), there is increasing evidence describing their role in both IIR and AIR modulation (MacDonald et al., 2011; Dalal et al., 2012). For instance, MSCs are able to not only inhibit NK proliferation and activity but also suppress T-/B-cell proliferation and DC maturation (Keating, 2012; Bruno et al., 2015). Furthermore, high levels of IFN-γ and TNF-α influence MSCs to develop immuno-suppressive properties toward IIR (affecting neutrophils, monocytes, and NKs) and AIR (T- and B-cells) (Corcione et al., 2006; Krampera et al., 2006; Spaggiari et al., 2006; Uccelli et al., 2008; Le Blanc and Mougiakakos, 2012). MSCs have also been reported to have a marked immuno-regulatory effect against autoimmune disorders and promising results have been observed in clinical trials on patients with Crohn' s disease and graft-vs.-host disease (Pittenger, 2009; Caplan and Correa, 2011).

It is still widely debated as to whether the immuno-modulatory action of MSCs relies on direct cell-to-cell interaction or on the paracrine action of soluble mediators released by these cells. Either one or both mechanisms are a possibility. Both, *in vivo* and *in vitro* studies have demonstrated that MSCs exhibit an immuno-suppressive role by suppressing T lymphocyte proliferation (Krampera et al., 2003; Aggarwal and Pittenger, 2005). A possible explanation for this role could be the presence of proteins and factors such as: TGF-β, hepatocyte growth factor (HGF), nitric oxide (NO), indoleamine 2,3-dioxygenase (IDO), human leukocyte antigen G (HLA-G), prostaglandin E2 (PGE-2), Inter Leukin (IL)-6, and IL-10 (Hwu et al., 2000; Aggarwal and Pittenger, 2005; Spaggiari et al., 2006; Djouad et al., 2007; Sato et al., 2007; English et al., 2009; Caplan and Correa, 2011) that are expressed highly by MSCs. For instance, It has been suggested, that IDO, PGE-2, and TGF-β1 are involved not only in the inhibition of NK cells but also in the inhibition of T-cell proliferation and activation (Aggarwal and Pittenger, 2005; Spaggiari et al., 2006). Furthermore, IL-6 has also been attributed to interfere with DC maturation (English et al., 2009). Finally, with a mechanism not completely known, MSCs have been reported to inhibit B-cells proliferation through the down-regulation of chemokine receptor CXCR4, CXCR5, and chemokine receptor type 7 (CCR7) (Corcione et al., 2006). The immuno-modulatory effects of MSCs can therefore be speculated to be achieved either through the release of the above mentioned factors and proteins directly into the extracellular milieu as soluble molecules, or they may be packaged into EVs together with nucleic acids and other post-transcriptional modulators which could influence the inflammatory response when released (Robbins and Morelli, 2014).

The ability of MSC-derived EVs to mimic the effect of the cell of origin has been studied on various different effector cells of the immune system (Blazquez et al., 2014; Conforti et al., 2014; Favaro et al., 2014, 2016; Zhang, 2014; Amarnath et al., 2015; Del Fattore et al., 2015; Gouveia de Andrade et al., 2015; Di Trapani et al., 2016; see Table 2).

**Table 2:** **The effect of stem cell derived EVs on the immune system**.

**Cell source**	**Target cell**	**EVs effect**	**References**
Human ASCs	T-lymphocytes	Down-regulation of T-cells proliferation	Blazquez et al., 2014
Human BM-MSCs	T-lymphocytes	Immuno-suppression through A2A receptor	Amarnath et al., 2015
Human BM-MSCs	T-lymphocytes	T regulatory cells increase	Del Fattore et al., 2015
		Increased release of IL-10	
Human BM-MSCs	B-lymphocytes	Down-regulation of B-cells proliferation	Conforti et al., 2014
		Down-regulation of B-cells differentiation	
		Inhibition of IgM, IgG and IgA production	
Human ESC-derived MSCs	Activated murine splenocytes	Splenocytes proliferation down-regulation	Zhang, 2014
		Switch to an M2-macrophage like phenotype	
		Increase of T regulatory cells *in vivo*	
Human BM-MSCs	PBMCs from type 1 diabetes patient	Down-regulation of Th1 mediated response	Favaro et al., 2014
		T regulatory cells increase	
		Th17 cells decrease	
Human BM-MSCs	Monocyte-derived DCs	Induction of regulatory DCs phenotype with inhibition of T-cell dependent immune response	Favaro et al., 2016
Human BM-MSCs	PBMCs, MSCs, NKs, B, and T-cells	Inhibition of NKs and B-cell proliferation	Di Trapani et al., 2016
		Increase of MSCs immunosuppressive properties	

BM, Bone Marrow; MSCs, Mesenchymal Stromal/Stem Cells; ESCs, Embryonic Stem Cells; PBMCs, Peripheral Blood Mononuclear Cells; ASCs, Adipose Stem Cells; DCs, Dendritic cells.

The inhibitory effect of MSC-derived EVs on the activation of cells of the immune system has been demonstrated by various studies. For example, Conforti et al. reported an inhibitory activity of MSCs and MSC-derived EVs on B-cell proliferation, which was further confirmed by a study conducted by Di Trapani et al. (Conforti et al., 2014). Del Fattore et al. also demonstrated that MSC-derived EVs not only increased the ratio between regulatory T-cells and effector T-cells, but also increased the release of the immunosuppressive cytokine IL-10, however, IDO an established mediator of MSC immunosuppressive effects was not affected (Del Fattore et al., 2015). A possible way by which MSC-EVs exhibit these immunomodulatory properties could be through an adenosine A2A receptor mediated mechanism (Amarnath et al., 2015). In addition, the extracellular environment could also play a role, for instance it has been reported that an inflammatory environment (mimicked in culture by the presence of IFN-γ and TNF-α) may not only alter the release but also influence the biological activity of MSC-derived EVs toward a more immunosuppressive role. In fact MSC-derived EVs may polarize monocytes toward M2-like phenotype, which in turn induces CD4^+^ T cell to differentiate into regulatory T cells (Zhang, 2014). A study by Blazquez et al. also reported that EVs derived from human adipose stem cells had an inhibitory effect not only on the differentiation and activation of T cells but also on the release of IFN-γ (Blazquez et al., 2014). In addition it has been shown that IFN-α or -γ transferred by EVs are also able to activate immunological molecular pathways into target cells (Li, 2013; Cossetti, 2014).

A study carried out by Favaro et al. looking at the effect of MSC-EVs on peripheral blood mononuclear cells (PBMCs) from type 1 diabetic patients revealed some interesting results. They showed that, MSC-derived EVs inhibited IFN-γ production in PBMCs stimulated by the islet Ag glutamic acid decarboxylase 65 (GAD65) and significantly increased the production of immune-modulatory mediators such as PGE-2, TGF-β, IL-10, and IL-6. In addition, there was a reduction in the number of Th17 cells as reflected by the reduction in IL17 secretion and an increase in regulatory T-cells in the same setting, suggesting that T-cells switched to an anti-inflammatory phenotype in the presence of EVs (Favaro et al., 2014). Furthermore, in a later study they reported that DCs conditioned with MSC-derived EVs acquired an immature phenotype with increased IL-10 secretion, which contributed toward inhibition of inflammatory T-cell response against islet Ags (Di Nicola et al., 2002; Favaro et al., 2014, 2016).

Although some very interesting studies have been reported in the literature explaining the immuno-modulatory functions of EVs on T-cell proliferation, there are some that are controversial. For instance, Conforti et al. and Gouveia de Andrade et al. both reported that the immuno-modulatory effects of EVs on T-cells are absent or significantly lower compared to MSCs (Conforti et al., 2014; Gouveia de Andrade et al., 2015). However, the apparent discrepancy observed in the different studies may depend on different methodological approaches and the activation state of the EV cell of origin. Recently Krampera's group attempted to standardize the method for studying the immunomodulatory effects of MSC-derived EVs, comparing unfractionated PBMCs with purified T, B, and NK cells (Di Trapani et al., 2016). In this study, they demonstrated a direct correlation between the degree of EV-mediated immuno-suppression and EV uptake by immune effector cells. In the case of PBMCs, most of the EVs were internalized by monocytes rather than by B and T cells. Furthermore, MSC-derived EVs did not significantly inhibit T-cell proliferation, whereas down-regulated the proliferation of NK and B-cells. These effects were enhanced by MSC-priming with inflammatory cytokines (Di Trapani et al., 2016). On investigating they observed that priming with MSCs increased the level of immunomodulatory miRNAs, such as miRNA-155 and miRNA-146 and that EVs obtained from primed MSCs potentiated the immunosuppressive properties of resting MSCs on T-cells with a mechanism that was independent to a direct EV inhibition of T-cell proliferation (Di Trapani et al., 2016).

Taken together these studies suggest that EVs derived from MSCs are less effective than the cells themselves, as the latter may act either by direct cell-to-cell interaction as well as by releasing several active soluble factors. Moreover, the biological effect of EVs may vary depending extracellular micro-environment. A pro-inflammatory environment for instance may modify the composition of EVs and the consequent biological activities as well as the activation state of immune effector cells targeted by these EVs.

## Potential therapeutic applications and concluding remarks

The ability of EVs to either enhance or suppress the immune response may be exploited in immuno-therapy. This dualistic effects of EVs makes them very flexible and lucrative, but, it also increases the risk of unpredictable adverse effects (Zhang et al., 2014). Several studies have reported how EV-dependent immuno-modulation can be correlated to the source type and the state of the cell of origin (activation and maturation degree). For example: APC and DC derived EVs can regulate Ag-specific and non-specific immune responses, both positively and negatively (Robbins and Morelli, 2014). The immunogenicity depends on the expression levels of MHC-class I and II, as well as co-stimulatory molecules such as CD80 and CD86 (Thomson and Robbins, 2008).

Although tumor-derived EVs seem to be mainly involved in immune-escape mechanisms, it has been demonstrated that APCs pulsed with EVs derived from tumor cells may cross-present Ags and stimulate the activation of an Ag-specific CTL-mediated anti-tumoral response (Escudier, 2005; Morse et al., 2005). This was also confirmed in a clinical trial conducted by Bu et al. on malignant gliomas (Bu et al., 2011). However, other clinical trials have also revealed the low immunogenicity of tumor-derived EVs, which represents the main limitation for this therapeutic strategy (Kunigelis and Graner, 2015).

The horizontal transfer of nucleic acid by EVs can be equally exploited in several therapeutic approaches (Robbins and Morelli, 2014). For example engineering EVs with miRNA or small interfering RNA (siRNA), capable of promoting or silencing the expression of particular transcripts, could be adopted in the future as therapeutic strategies.

Immune-cell-based and stem cell based therapies currently present several risks and complications as well as technical problems such: culture-induced senescence, immune-mediated rejection, genetic instability, loss of functional properties, and malignant transformation that need to be solved to make them successful (Nawaz et al., 2016). EVs which mimic in part the effect of the stem cells from which they are derived may represent a novel cell-free solution, which could overcome some of the limitations mentioned above linked to cell based therapies (Zhang, 2014). Giebel et al. in a case report demonstrated the feasibility of an *in vivo* EV approach for the therapy of refractory graft vs. host disease (Kordelas et al., 2014). The results of this study suggested a beneficial effect of MSC-derived EVs as anti-inflammatory and immune-modulatory mediators in the context of a severe inflammatory and immune disease. Nevertheless, in order to develop an effective and successful immuno-therapy based on stem cell derived EVs warrants not only further studies to clarify the mechanism of action of EVs, but also the standardization of protocols for isolation and characterization (Witwer et al., 2013).

## Author contributions

All authors listed, have made substantial, direct and intellectual contribution to the work, and approved it for publication.

### Conflict of interest statement

The authors declare that the research was conducted in the absence of any commercial or financial relationships that could be construed as a potential conflict of interest.

## Abbreviations

EVs: Extra-cellular vesicles
Ag(s): Antigen(s)
miRNA: MicroRNA
APCs: Antigen presenting cells
DCs: Dendritic cells
NKs: Natural killer cells
IIR: Innate immune response
AIR: Acquired immune response
TGF-β: Transforming Growth factor-β
CTLs: Cytotoxic T-lymphocytes
RA: Rheumatoid arthritis
SC(s): Stem cell(s)
ESCs: Embryonic stem cells
MSCs: Mesenchymal stem/stromal cells
iPS: Induced pluripotent stem cells
IFN-γ: Interferon-γ
TNF-α: Tumor Necrosis Factor-α
HGF: Hepatocyte Growth Factor
NO: Nitric oxide
IDO: Indoleamine 2,3-dioxygenase
HLA-G: Human Leukocyte Antigen-G
PG: Prostaglandin
IL: Interleukin
CXCR4/CXCR5/CCR7: Chemokine Receptor Type 4/5–7
PBMCs: Peripheral Blood Mononuclear Cells
GAD65: Glutamic Acid Decarboxylase 65
NKG2D: Natural-Killer Group-2 Member-D receptor
MHC: Major Histocompatibility Complex
siRNA: Small interfering RNA
BM: Bone Marrow
ASCs: Adipose Stem Cells.
